# Co‐occurrence of schwannomatosis and rhabdoid tumor predisposition syndrome 1

**DOI:** 10.1002/mgg3.412

**Published:** 2018-05-20

**Authors:** Hildegard Kehrer‐Sawatzki, Uwe Kordes, Simone Seiffert, Anna Summerer, Christian Hagel, Ulrich Schüller, Said Farschtschi, Reinhard Schneppenheim, Martin Bendszus, Tim Godel, Victor‐Felix Mautner

**Affiliations:** ^1^ Institute of Human Genetics University of Ulm Ulm Germany; ^2^ Department of Pediatric Hematology and Oncology University Medical Center Hamburg‐Eppendorf Hamburg Germany; ^3^ Institute of Neuropathology University Medical Center Hamburg‐Eppendorf Hamburg Germany; ^4^ Research Institute Children's Cancer Center Hamburg Hamburg Germany; ^5^ Department of Neurology University Medical Center Hamburg‐Eppendorf Hamburg Germany; ^6^ Department of Neuroradiology University of Heidelberg Medical Center Heidelberg Germany

**Keywords:** atypical teratoid/rhabdoid tumor, deletion breakpoint, RTPS1, schwannomatosis, *SMARCB1* gene

## Abstract

**Background:**

The clinical phenotype associated with germline *SMARCB1* mutations has as yet not been fully documented. It is known that germline *SMARCB1* mutations may cause rhabdoid tumor predisposition syndrome (RTPS1) or schwannomatosis. However, the co‐occurrence of rhabdoid tumor and schwannomas in the same patient has not so far been reported.

**Methods:**

We investigated a family with members harboring a germline *SMARCB1* deletion by means of whole‐body MRI as well as high‐resolution microstructural magnetic resonance neurography (MRN). Breakpoint‐spanning PCRs were performed to characterize the *SMARCB1* deletion and its segregation in the family.

**Results:**

The index patient of this family was in complete continuous remission for an atypical teratoid/rhabdoid tumor (AT/RT) treated at the age of 2 years. However, at the age of 21 years, she exhibited paraparesis of her legs and MRI investigations revealed multiple intrathoracic and spinal schwannomas. Breakpoint‐spanning PCRs indicated that the germline deletion segregating in the family encompasses 6.4‐kb and includes parts of *SMARCB1* intron 7, exons 8–9 and 3.3‐kb located telomeric to exon 9 including the *SMARCB1* 3′ UTR. The analysis of sequences at the deletion breakpoints showed that the deletion has been caused by replication errors including template‐switching. The patient had inherited the deletion from her 56‐year‐old healthy mother who did not exhibit schwannomas or other tumors as determined by whole‐body MRI. However, MRN of the peripheral nerves of the mother's extremities revealed multiple fascicular microlesions which have been previously identified as indicative of schwannomatosis‐associated subclinical peripheral nerve pathology.

**Conclusion:**

The occurrence of schwannomatosis‐associated clinical symptoms independent of the AT/RT as the primary disease should be considered in long‐term survivors of AT/RT. Furthermore, our investigations indicate that germline *SMARCB1* mutation carriers not presenting RTs or schwannomatosis‐associated clinical symptoms may nevertheless exhibit peripheral nerve pathology as revealed by MRN.

## 1 INTRODUCTION

Rhabdoid tumors (RTs) are aggressive malignancies termed atypical teratoid (AT)/RTs when they arise in the central nervous system, malignant rhabdoid tumor (MRT) when located in extra‐renal soft tissues or RT of the kidney (RTK). The majority of RTs are caused by biallelic loss of function of the *SMARCB1* gene located at 22q11.23 (Biegel et al., 1999; Versteege et al., 1998). Approximately 30% of patients with RTs exhibit germline *SMARCB1* mutations causing RT predisposition syndrome 1 (RTPS1, OMIM #609322) (Bourdeaut et al., 2011; Eaton, Tooke, Wainwright, Judkins, & Biegel, 2011; Janson et al., 2006; Kordes et al., 2010; Sévenet et al., 1999). So far, six families have been reported with siblings affected by RTs due to the transmission of a *SMARCB1* mutation from a healthy parent, with gonadal mosaicism for the respective *SMARCB1* mutation (Bourdeaut et al., 2011; Bruggers et al., 2011; Eaton et al., 2011; Gigante et al., 2016; Sévenet et al., 1999).

Germline mutations in *SMARCB1* also cause schwannomatosis (OMIM #162091). It has been shown that 48% of familial and 9.8% of sporadic patients with schwannomatosis exhibit germline *SMARCB1* mutations (Boyd et al., 2008; Hadfield et al., 2008; Hulsebos et al., 2007; Rousseau, Noguchi, Bourdon, Sobol, & Olschwang, 2011; Sestini, Bacci, Provenzano, Genuardi, & Papi, 2008; Smith, Wallace, Bowers, Eaton, & Evans, 2014; Smith et al., 2012). Genetic heterogeneity is observed in schwannomatosis since germline mutations in *LZTR1* have also been identified in patients with the disease (Hutter et al., 2014; Louvrier et al., 2018; Paganini et al., 2015; Piotrowski et al., 2012; Smith et al., 2015). Furthermore schwannomatosis predisposition genes are likely to exist since germline mutations in *SMARCB1* or *LZTR1* are not detectable in at least 50% of sporadic patients with schwannomatosis (Kehrer‐Sawatzki, Farschtschi, Mautner, & Cooper, 2017). However, the frequency of somatic mosaicism of *SMARCB1* or *LZTR1* mutations in patients with schwannomatosis is as yet unclear. Schwannomatosis is characterized by the occurrence of multiple schwannomas most commonly affecting the peripheral nerves (89%) and the spine (74%) (Merker, Esparza, Smith, Stemmer‐Rachamimov, & Plotkin, 2015). The incidence of schwannomatosis is still unclear, but most sources suggest an incidence comparable to that of NF2 (Antinheimo et al., 2000; Koontz et al., 2013). Schwannomatosis is most commonly diagnosed during adulthood. Merker et al. (2015) analyzed 87 patients with schwannomatosis and reported a median age at initial symptom presentation of 30 years (with a range between 8 and 59 years) and a median age at diagnosis of 40 years (with a range between 16 and 70 years). Schwannomas are benign nerve sheath tumors that generally do not transform toward malignancy (Carter et al., 2012). In contrast, the vast majority of AT/RTs are tumors of infancy and early childhood with a poor prognosis.

At least 30% of patients with RTs have germline *SMARCB1* mutations which are mostly de novo (Bourdeaut et al., 2011; Eaton et al., 2011; Kordes et al., 2010). Approximately one‐third of patients with RTs and germline *SMARCB1* mutations inherited the mutation from a parent who also harbors the mutation in his or her blood cells (Eaton et al., 2011). So far, only four families have been reported, with germline *SMARCB1* mutation carriers being either affected by schwannomatosis or RT (Table 1) (Carter et al., 2012; Eaton et al., 2011; Sredni & Tomita, 2015; Swensen et al., 2009). The co‐occurrence of RT and schwannomatosis in the same patient has however not been reported as yet. AT/RT is often associated with a poor prognosis and early death but long‐term survival is possible by means of intensive multimodal treatment (Ammerlaan et al., 2008; Bartelheim et al., 2016; Kordes et al., 2014; Squire, Chan, & Marcus, 2007; Tekautz et al., 2005). Favorable prognostic factors are inheritance of the *SMARCB1* germline mutation from a healthy carrier of the mutation, absence of synchronous tumors and older age at presentation (Bartelheim et al., 2016; Kordes et al., 2014).

**Table 1:** Familial *SMARCB1* mutations and family members affected by schwannomatosis or rhabdoid tumor (RT) as previously published

Number of *SMARCB1* mutation carriers and clinical details	*SMARCB1* germline mutation	Reference
Four generations of the family were affected. Six family members were mutation carriers; three of them had schwannomatosis, two died from RT as infants and one mutation carrier was clinically unaffected. However, MRI investigations were not performed	Direct duplication of 2,631‐bp including parts of intron 5 and 6, as well as complete exon 6 causing a frameshift and protein truncation (p.Leu266fs)	Swensen et al. (2009)
The female mutation carrier was affected by RT. Her father and paternal grandmother were also mutation carriers and had schwannomatosis but not RT	c.472C>T, exon 4	Eaton et al. (2011)
The mother carried the mutation and had schwannomatosis. One of her schwannomas transformed to an epithelioid malignant peripheral nerve sheath tumor. All three of her children harbored the mutation, two of whom had RT, whereas one was asymptomatic	Frameshift mutation c.245_246insAT, exon 3	Carter et al. (2012)
Two sisters exhibited RT, while their father and paternal grandmother had schwannomatosis	Not investigated	Sredni & Tomita (2015)

**Figure 1:**
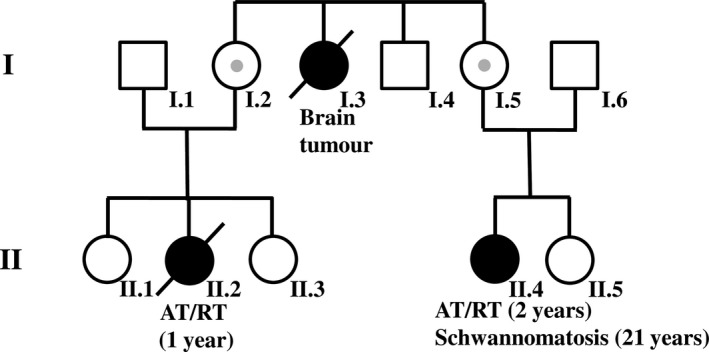
Pedigree of the family investigated in this study. Individuals II.2 and II.4 had atypical teratoid/rhabdoid tumor (AT/RT). Patient II.4 underwent surgery at the age of two years to resect an AT/RT located in the posterior fossa. Patient II.2 died at the age of one year owing to complications at primary surgery of a *SMARCB1*‐negative AT/RT. The germline *SMARCB1* deletion was detected in patient II.4, her mother and was also in the AT/RT of patient II.2. Consequently, proband I.2 also carries the germline *SMARCB1* deletion of exons 8–9

In this study, we report the follow‐up of a patient (II.4, Figure 1) with a germline *SMARCB1* deletion of exons 8–9 who survived an AT/RT treated at the age of 2 years without tumor recurrence as determined by analysis of the patient at the age of 17 years (Kordes et al., 2014). She had inherited the germline *SMARCB1* deletion from her healthy mother, proband I.5 (Figure 1). In this study, we analyzed the *SMARCB1* deletion segregating in the family of patient II.4 in greater detail and report on the subsequent course of the disease in patient II.4 who was diagnosed with schwannomatosis at the age of 21 years. We used whole‐body and cranial magnetic resonance imaging (MRI) to investigate the tumor load as well as high‐resolution microstructural magnetic resonance neurography (MRN) to quantify peripheral nerve pathology at the fascicular level in patient II.4 as well as in her clinically unaffected mother I.5.

## 2 PATIENTS AND METHODS

The patients provided written informed consent and the study was approved by the institutional review boards.

### 2.1 Whole‐body MRI examination

Patient II.4 and her mother I.5 were investigated by means of whole‐body MRI using a Magnetic Resonance scanner (Magnetom SKYRA; Magnetom VERIO and Magnetom TIM TRIO, Siemens Healthineers) at 3 Tesla with T1‐ and T2‐weighted sequences according to standard protocols in order to determine the occurrence of tumorous lesions >1 cm in diameter.

### 2.2 High‐resolution, microstructural peripheral nerve MRI

Large coverage, high‐resolution, microstructural peripheral nerve MRI also termed microstructural magnetic resonance neurography (MRN) was performed at 3‐Tesla in order to investigate the presence of small fascicular nerve lesions in the extremities of patient II.4 and her clinically unaffected mother I.5. Contiguous coverage of upper and lower extremities was achieved by applying an array of fat‐saturated T2‐weighted high‐resolution sequences as described previously (Bäumer et al., 2013). Quantitative analysis was performed by the evaluation of cross‐sectional images. The fascicular nerve lesions were categorized as non‐compressive fascicular microlesions (<2 mm in diameter), intermediate lesions (2–5 mm in diameter) and compressive macrolesions (≥5 mm in diameter).

### 2.3 Microarray analysis

CytoScan HD arrays (Affymetrix) were used to investigate blood‐derived genomic DNAs from patient II.4 and her mother I.5 following the manufacturer's protocols and instructions. The array consists of 2,696,550 probes that include 743,304 SNPs and 1,953,246 non‐polymorphic probes. The Affymetrix Chromosome Analysis Suite (ChAS) software was used to detect and evaluate copy‐number variants (CNVs).

### 2.4 Breakpoint‐spanning PCRs

Long‐range breakpoint‐spanning PCRs were performed using the Expand™ Long Range dNTPack (Merck) which contains a mixture of Taq polymerase and a thermostable DNA polymerase with proof reading activity optimized to efficiently amplify long PCR products. The primers used to amplify breakpoint‐spanning PCR products are listed in Supporting Information Table S1 (*SMARCB1* GenBank accession number: NG_009303.1). In addition, we performed short breakpoint‐spanning PCRs with primers listed in Supporting Information Table S2 using the AmpliTaq Gold™ polymerase (Thermo Fisher Scientific) and DNA isolated from paraffin‐embedded AT/RT tissue from patient II.2 or DNA isolated from fresh‐frozen schwannoma tissue (T2763) of patient II.4. PCR products were sequenced using the BigDye™ Terminator v3.1 Cycle Sequencing Kit (Thermo Fisher Scientific) and an ABI 3130 genetic analyzer (Applied Biosystems).

### 2.5 Mutation analysis

Blood‐derived genomic DNA of patient II.4 as well as genomic DNA isolated from fresh‐frozen schwannoma tissue of patient II.4 were analyzed by PCR and Sanger sequencing of the respective PCR products in order to identify mutations within the *NF2* and *LZTR1* genes (GenBank accession numbers: NG_009057.1; NG_034193.1). The primers used for these analyses are listed in Supporting Information Tables S3 and S4. The DNA samples were also investigated by MLPA in order to identify intragenic copy‐number variants within *NF2* and *LZTR1* by means of the SALSA MLPA probemix P455 *LZTR1* (version A1) and the SALSA MLPA P044 *NF2* probemix (version B3; MRC Holland, The Netherlands).

### 2.6 Analysis of the sequences flanking the *SMARCB1* deletion breakpoints

Genomic regions immediately flanking the germline *SMARCB1* deletion were investigated for the presence of sequences that may adopt alternative conformations such as non‐B DNA using the non‐B DB database (https://nonb-abcc.ncifcrf.gov/apps/nBMST/default/) (Cer et al., 2013).

## 3 RESULTS

### 3.1 Clinical findings

#### 3.1.1 Patient II.4

**Figure 2:**
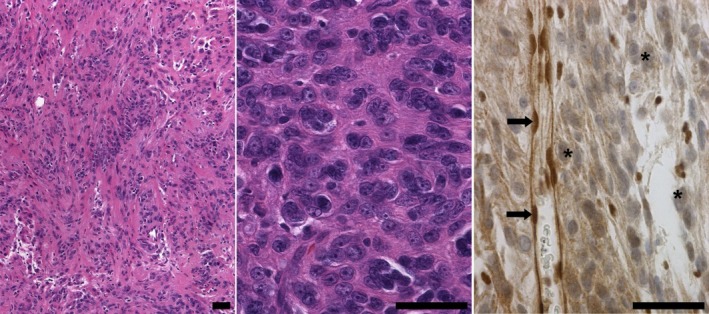
Histopathological investigation of the schwannoma (T2763) of patient II.4 revealed palisading of nuclei typical for schwannomas (left, H&E) and areas of high cellularity and rhabdoid features comprising round, sharply demarcated cells with eccentric nuclei and large nucleoli (middle, H&E). Immunohistochemical staining with the INI1/BAF47 antibody (BD Transduction Laboratories) indicated loss of nuclear SMARCB1 expression in most tumor cells (asterisk) but not in endothelial cells (arrow). Scale bars: 50 μm

Female patient II.4 underwent surgery at the age of 2 years to resect an AT/RT located in the posterior fossa. The tumor was diagnosed by immunohistochemistry as a *SMARCB1/INI* negative AT/RT (Kordes et al., 2014). After high‐dose chemotherapy and autologous stem cell rescue she survived and was without evidence of malignancy recurrence at the age of 17 years as confirmed by MRI (Kordes et al., 2014). In the present study, we report on the clinical follow‐up of patient II.4. At the age of 21 years, the patient exhibited mild aconuresis, paraparesis of both legs and progressive gait disturbances present since 4 months. MRI analysis of the patient revealed a large intraspinal schwannoma (T2763) with paraspinal extension located in the region of the thoracic vertebral bodies 8 and 9. The tumor encompassed 5.3 × 3.6 cm and caused profound compression of the spinal cord. It was removed by surgery when the patient was 21 years of age and her clinical symptoms, including aconuresis and paraparesis, improved considerably afterward. The tumor was classified by means of histopathological examination as a schwannoma (WHO grade I) with rhabdoid features (Figure 2).

Whole‐body MRI of patient II.4 exhibited additional intraabdominal, intrathoracic and spinal T2‐hyperintensive nodules of 2–3 cm in diameter suggestive of schwannomas. The nodules were located paravertebrally along the thoracic and the lumbar spine, within the intercostal and pelvic regions, the left pectoral girdle and left upper arm. Multiple T2‐hyperintense neurogenic nodules up to 3 cm in diameter were observed in both lower legs located intramuscular and along the major nerve trunks.

**Figure 3:**
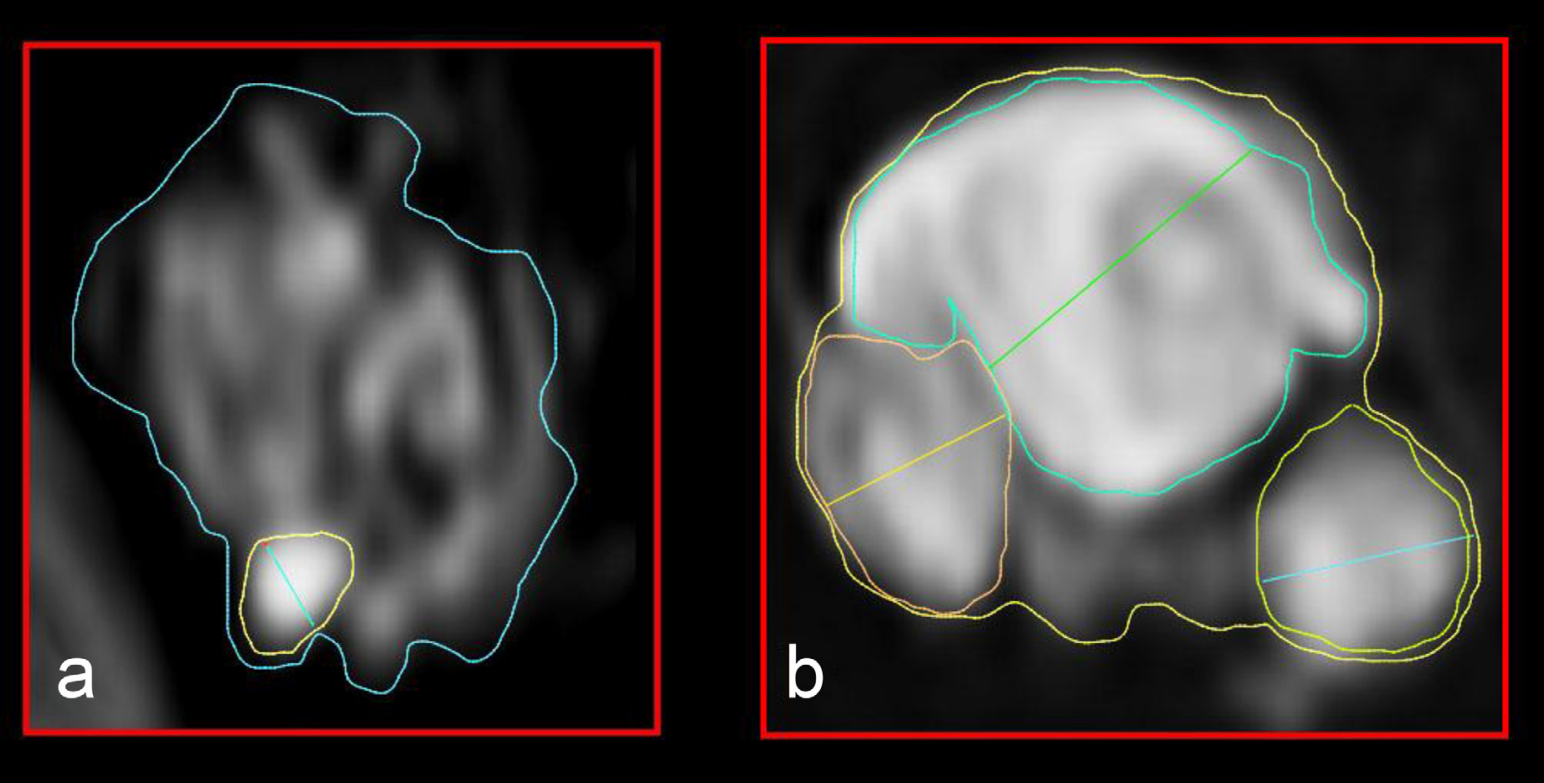
Microstructural images of fascicular T2 lesions of the sciatic nerve. (a) In the clincially unaffected mother (proband I.5), microstructural magnetic resonance neurography (MRN) revealed a non‐compressive fascicular microlesion (<2 mm in diameter). (b) Intermediate fascicular lesions (2–5 mm) and macrolesions (>5 mm) were detected in patient II.4

By means of high‐resolution, microstructural peripheral nerve MRI of the extremities, multiple fascicular microlesions (<2 mm), intermediate lesions (2–5 mm) and macrolesions (≥5 mm) were detected (Table 2, Figure 3). Cranial MRI performed at the age of 21 years did not indicate RT relapse or other brain tumors.

**Table 2:** Number of fascicular peripheral nerve lesions observed by high‐resolution microstructural nerve MRI in patient II.4 and her mother I.5

Individual investigated	Extremity	Affected nerve	Number of fascicular		
			IMicrolesions (2–5 mm)	Intermediate lesions (2–5 mm)	Macrolesions (≥5 mm)
Patient II.4	Proximal left thigh	Sciatic	6	5	6
	Distal left thigh	Tibial	2	–	5
	Distal left thigh	Peroneal	3	1	–
	Left lower leg	Tibial	5	5	1
	Proximal right thigh	Sciatic	5	1	1
	Distal right thigh	Tibial	4	–	1
	Distal right thigh	Peroneal	1	–	–
	Right lower leg	Tibial	4	3	1
	Left upper arm	Median	3	–	–
	Left upper arm	Ulnar	1	–	–
	Left upper arm	Radial	4	1	1
	Right upper arm	Median	7	–	–
	Right upper arm	Ulnar	5	–	–
	Right upper arm	Radial		–	–
Proband I.5	Proximal left thigh	Sciatic	2	–	–
	Distal left thigh	Tibial	2	–	–
	Distal left thigh	Peroneal	2	–	–
	Left lower leg	Tibial	3	2	–
	Proximal right thigh	Sciatic	1	–	–
	Distal right thigh	Tibial	1	–	–
	Distal right thigh	Peroneal	1	–	–
	Right lower leg	Tibial	2	–	–
	Left upper arm	Median	–	–	–
	Left upper arm	Ulnar	2	–	–
	Left upper arm	Radial	1	–	–
	Right upper arm	Median	1	–	–
	Right upper arm	Ulnar	4	–	–
	Right upper arm	Radial	–	–	–

–, none detected.

#### 3.1.2 Mother of patient II.4

The mother of patient II.4 (proband I.5, Figure 1) did not exhibit any clinical symptoms of schwannomatosis at the age of 56 years. Whole‐body and cranial MRI investigation did not indicate any tumorous T2‐hyperintense lesions. We also performed high‐resolution, microstructural peripheral nerve MRI to detect small fascicular abnormalities in the nerves which are not detectable by conventional MRI. These investigations did not indicate the presence of compressive macrolesions (≥5 mm) along the peripheral nerves of her extremities. However, we detected non‐compressive fascicular microlesions (<2 mm) of the peripheral nerves of all four extremities and two intermediate lesions (2–5 mm) in the left lower leg indicative of peripheral nerve pathology in proband I.5 (Table 2). Taken together, 11 microlesions were detected in her left leg and five microlesions in her right leg.

In contrast, microlesions are very rare in healthy control individuals. Previous analyses indicated microlesions in only three of 25 healthy control individuals investigated (Farschtschi et al., 2016). Furthermore, the three control individuals exhibited microlesions only in the lower extremities. On average, 3.5 microlesions were detected per extremity in these three controls (Farschtschi et al., 2016). This number of microlesions is considerably lower than the number of microlesions detected in proband I.5. Remarkably, the 25 healthy controls analyzed previously by means of MRN did not exhibit microlesions in the upper extremities (Farschtschi et al., 2016). In contrast to this, proband I.5 exhibited three microlesions in the left upper arm and five microlesions in the right upper arm.

### 3.2 Molecular findings

#### 3.2.1 Identification of the *SMARCB1* germline deletion breakpoints

**Figure 4:**
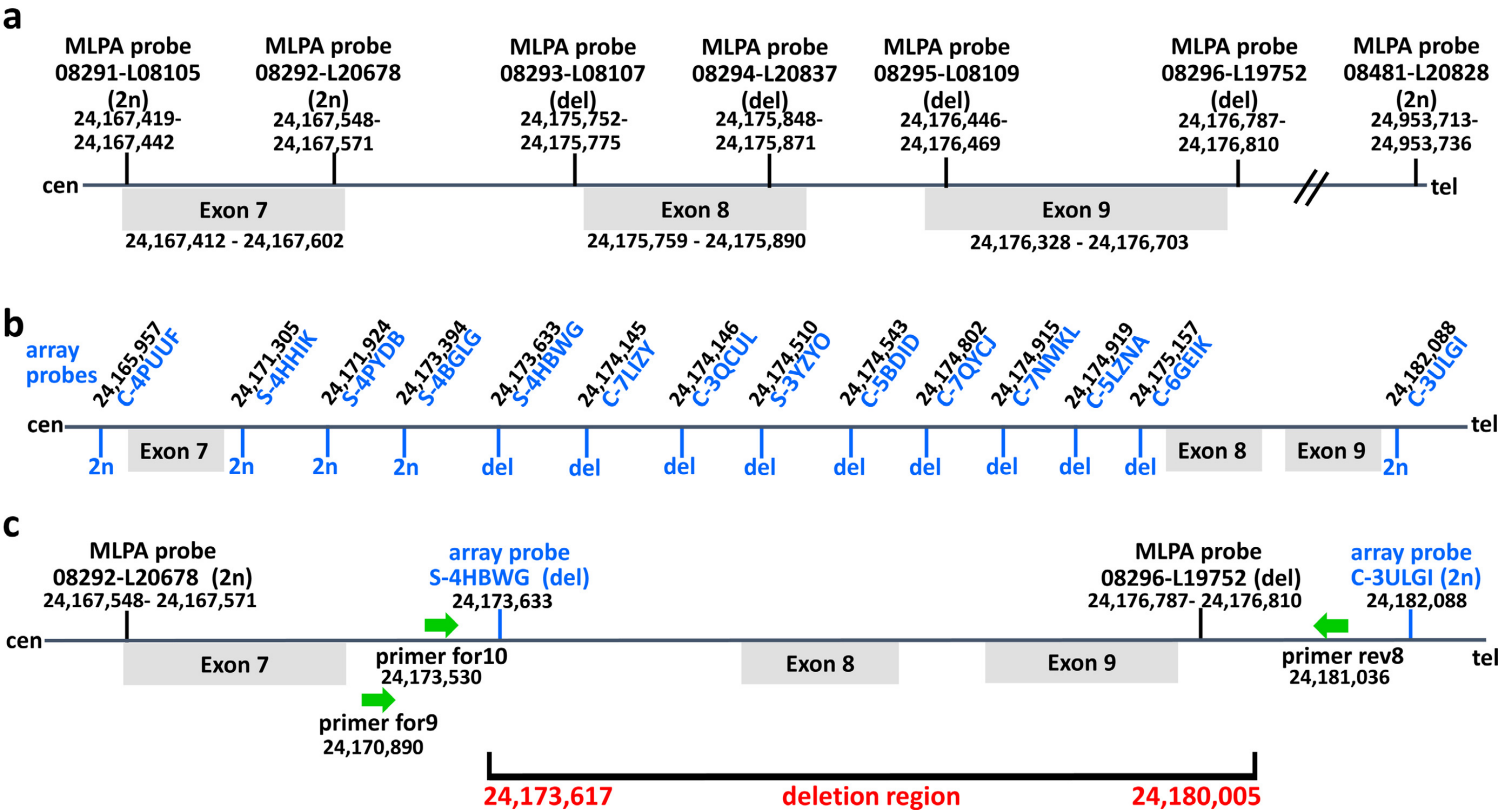
Schema indicating the results of the MLPA and the CytoScan HD array analyses performed to narrow down the breakpoints of the *SMARCB1* germline deletion identified in the family studied here. According to these results, breakpoint‐spanning PCRs were performed to identify the breakpoints at highest resolution. (a) The genomic positions of the MLPA probes are indicated as well as the relative locations of the *SMARCB1* exons. (b) The relative positions of the array probes as well as their genomic locations are presented in this part of the schema. MLPA and array probes indicating the loss of one copy in the patient are labeled as “del” whereas those indicating diploid copy numbers are labeled as “2n”. (c) Breakpoint‐spanning PCRs performed with primers represented as green arrows revealed that the deletion region encompasses 6,388‐bp and involves parts of *SMARCB1* intron 7, complete exon 8, intron 8, exon 9 and 3,302‐bp located telomeric to exon 9. The genomic positions indicated are according to the human genome reference sequence (hg19)

The *SMARCB1* deletion detected in patient II.4 and her mother I.5 includes exons 8 and 9 as previously determined by MLPA (Kordes et al., 2014). In the present study, we characterized the deletion in greater detail. We first performed CytoScan HD array analysis using blood‐derived genomic DNA of patient II.4 in order to narrow down the deletion breakpoint regions (Figure 4). Subsequently, we performed breakpoint‐spanning PCRs with primers US1_for10 and rev8 (Figure 4c, Supporting Information Figure S1). Sequence analysis of the 1,138‐bp breakpoint‐spanning PCR product identified the deletion junction indicating that the deletion encompasses 6,388‐bp. The deletion includes parts of *SMARCB1* intron 7, exons 8–9 and 3,302‐bp located telomeric to exon 9 including the 3′ UTR of *SMARCB1* as well as the 3′‐end of the *DERL3* gene which flanks *SMARCB1* in a telomeric direction. A breakpoint‐spanning PCR product was also obtained using primers for9 and rev8 (Supporting Information Figure S2). Both breakpoint‐spanning products (for9/rev8 and US1_for10/rev8) were also amplified from genomic DNA of the mother of patient II.4, proband I.5. Sequence analyses of these breakpoint‐spanning PCR products did not indicate any sequence differences between patient II.4 and her mother within 3,756‐bp flanking the deletion breakpoints.

#### 3.2.2 Mechanism causing the germline *SMARCB1* deletion

**Figure 5:**
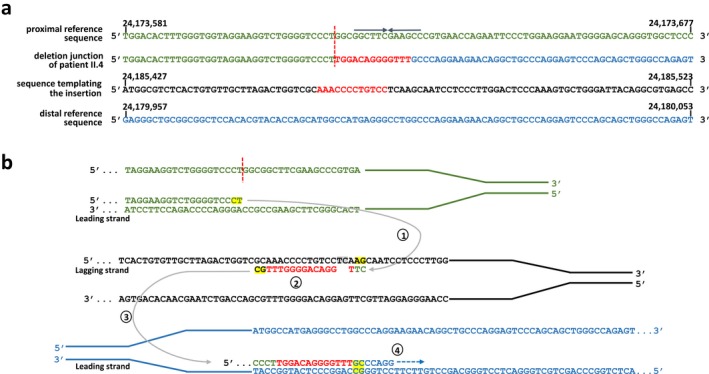
The germline *SMARCB1* deletion investigated in this study, as well as the insertion of 13‐bp (red) at the deletion breakpoints, most likely resulted from replication‐associated template switching. (a) Alignment of the deletion breakpoint‐flanking sequences of patient II.4 against the reference sequence of the human genome (hg19). Sequences located at the proximal (centromeric) deletion breakpoint are indicated in green, while sequences at the distal (telomeric) breakpoint are given in blue. The vertical red line highlights the position of the proximal deletion breakpoint. The 13‐bp insertion (red) identified at the deletion junction exhibits homology to a sequence located 5.5‐kb telomeric to the distal breakpoint region. (b) Model proposed to explain the origin of the deletion‐associated insertion. In the proximal breakpoint‐flanking region, DNA synthesis at the leading strand is interrupted but appears to have resumed, after an interstrand template switch, to a replication fork located 5.5‐kb telomeric to the distal breakpoint‐flanking region (step 1). Subsequently, the 13‐bp indicated in red are newly synthesized and included in the nascent DNA strand at the replication fork (step 2). Single‐nucleotide changes due to DNA polymerase errors are highlighted in gray. Subsequently, another template switch occurs on the leading strand (step 3) upon which replication is continued (step 4). The nucleotides exhibiting microhomology at sites of template switching are marked in yellow. An inverted repeat of 6‐bp marked by arrows was identified close to the proximal deletion breakpoint which may have caused a cruciform structure responsible for replication stalling

Neither the proximal deletion breakpoint in *SMARCB1* intron 7 nor the distal deletion breakpoint located 3′ to the *SMARCB1* gene were situated within high‐copy repeat sequences such as *ALU*‐ or L1‐elements. Furthermore, extended sequence homology between proximal and distal breakpoint regions was not observed. We conclude that the deletion has not been mediated by a mechanism dependent upon extended sequence similarity at the breakpoints. A 13‐bp insertion was identified at the deletion junction, which may have been templated by a sequence located 5.5‐kb telomeric to the distal deletion breakpoint (Figure 5). Hence, a template‐switching mechanism during replication such as fork stalling and template switching (FoSTeS), microhomology‐mediated break‐induced replication (MMBIR) or serial replication slippage has most likely caused the *SMARCB1* deletion. Indeed, DNA replication‐based mechanisms are frequently causing disease‐associated multi‐exon copy number variants as well as polymorphic structural variants in the human genome (Abyzov et al., 2015; Ankala et al., 2012; Hsiao et al., 2015; Zhang et al., 2009, 2010). We identified a short inverted repeat located close to the proximal deletion breakpoint which may have templated the formation of a cruciform structure responsible for replication stalling (Figure 5). Short inverted repeats are known to induce DNA instability mediated by replication‐related mechanisms (Lu et al., 2015).

#### 3.2.3 Segregation of the germline *SMARCB1* deletion in other family members

A maternal niece (patient II.2, Figure 1) had died at the age of one year due to complications at primary surgery of a *SMARCB1*‐negative AT/RT, but genetic analysis was not performed (Kordes et al., 2014). In the present study, we analyzed DNA isolated from paraffin‐embedded AT/RT tissue of patient II.2 by means of breakpoint‐spanning PCR and confirmed the presence of the germline *SMARCB1* deletion (Supporting Information Figure S3). This finding indicates the segregation of the *SMARCB1* germline deletion in the family and that proband I.2 must be an obligate carrier of the deletion (Figure 1). Analysis of proband I.2 was however not possible since she declined clinical or genetic investigations.

#### 3.2.4 Genetic analysis of a schwannoma from patient II.4

Germline mutations within *LZTR1* or *NF2* were neither detected in blood‐derived DNA of patient II.4 nor in genomic DNA isolated from schwannoma T2763 of patient II.4. MLPA analysis of schwannoma‐derived DNA indicated the somatic loss of one copy of *LZTR1* and *NF2* which was not detected in the patient's blood. Our findings imply that a large tumor‐specific deletion had occurred on the chromosome 22 not harboring the germline *SMARCB1* deletion of exons 8–9. This somatic large deletion includes one allele of *LZTR1* and *NF2* as well as the wild‐type allele of *SMARCB1* (Supporting Information Figure S5). We were able to conclude this from the observation that the *SMARCB1* exon 8–9 deletion breakpoint‐spanning PCR fragment was strongly amplified from DNA of schwannoma T2763 but not *SMARCB1* exons 8 and 9. Hence, most of the tumor cells exhibit biallelic loss of *SMARCB1* exons 8 and 9.

## 4 DISCUSSION

Atypical teratoid/RTs are brain tumors most commonly affecting children younger than 3 years of age (Hilden et al., 2004; Rorke, Packer, & Biegel, 1996). AT/RTs are predominantly infratentorial malignancies, most of them located within the posterior fossa. However, AT/RTs may also be located in supratentorial brain regions (Dho et al., 2015). AT/RT caused by the complete loss of function of *SMARCB1* is often associated with a poor prognosis and early death but long‐term survival is possible in some cases (Ammerlaan et al., 2008; Bartelheim et al., 2016; Kordes et al., 2014; Squire et al., 2007; Tekautz et al., 2005). In this study, we report the follow‐up of female patient II.4 with a germline *SMARCB1* deletion who survived an AT/RT surgically removed at the age of 2 years (Kordes et al., 2014). Patient II.4 is the first case documented to be in complete continuous remission for a AT/RT but diagnosed with schwannomatosis during adulthood. Our findings indicate that schwannomatosis‐associated tumorigenesis and related clinical symptoms need to be taken into account in patients surviving RTs and harboring germline *SMARCB1* mutations.

Reinvestigation of the *SMARCB1* deletion previously identified in patient II.4 indicated that the deletion encompasses 6.4‐kb and parts of *SMARCB1* intron 7, exons 8–9. Analysis of the breakpoint sequences implies that the deletion has been mediated by a mutational mechanism involving replication‐associated template‐switching (Figure 5). The mother of patient II.4 also carries the *SMARCB1* deletion. Sequence analysis of regions flanking the deletion breakpoints did not indicate any sequence differences between the mother and her daughter. By means of deletion junction‐spanning PCR, we were able to demonstrate that the niece of proband I.5 and the sister of proband I.5 (individuals II.2 and I.2, Figure 1) are both carriers of the germline *SMARCB1* deletion.

Probands I.2 and I.5 were not affected by AT/RT as children even although they are carriers of the germline *SMARCB1* deletion. The risk of RT development seems to be time‐dependent in the sense that a specific developmental time window exists during which the tumor progenitor cell is vulnerable to complete SMARCB1 protein loss initiating RT growth. If this sensitive period is completed without biallelic *SMARCB1* inactivation, the growth of an AT/RT is not initiated. This hypothesis has been supported by tissue and developmental stage‐specific conditional knockout mouse models which showed that biallelic *Smarcb1* loss in early neural crest cells (embryonic day 9.5) initiates RT growth in the cranial nerves and meninges. After embryonic day 12.5, inactivation of *Smarcb1* in peripheral Schwann cells or in brain glial cells does not initiate RTs (Vitte, Gao, Coppola, Judkins, & Giovannini, 2017). Hence, an early spatio‐temporal window must exist during which *Smarcb1* loss results in malignant transformation of specific neural crest cells. The absence of AT/RT in *SMARCB1* mutation carriers is thus explained by retention of the *SMARCB1* wild‐type allele and its activity in neural crest cells during early stages of embryonic development.

It is however surprising that the mother of patient II.4 (proband I.5) does not show clinical symptoms of schwannomatosis even though she also carries the germline *SMARCB1* deletion. Whole‐body MRI and cranial MRI investigations of proband I.2 did not indicate any tumorous T2‐hyperintense lesions. However, high‐resolution, microstructural peripheral nerve MRI, also termed microstructural magnetic resonance neurography (MRN) revealed non‐compressive fascicular microlesions (<2 mm) and intermediate lesions (2–5 mm) along the peripheral nerves of the extremities in proband I.5 (Table 2; Figure 3). Intrafascicular microlesions have previously been shown to represent frequent features of peripheral nerve pathology in patients with schwannomatosis (Farschtschi et al., 2016). It is however unclear as yet whether these microlesions are predictive of future symptomatic tumors. Our results nevertheless imply that microstructural MRN is a method well suited to detecting subclinical peripheral nerve pathology in clinically unaffected *SMARCB1* mutation carriers such as proband I.5. According to the recommended surveillance protocols for patients with AT/RT and truncating germline *SMARCB1* mutations, whole‐body MRI should be considered up to the age of 5 years (Foulkes et al., 2017). Our findings suggest that long‐term survivors of AT/RT and truncating germline *SMARCB1* mutations should receive regular surveillance for schwannomatosis‐associated clinical symptoms.

## CONFLICT OF INTEREST

The authors declare that they have no conflict of interest.
