# Supplementary material for: Co‐occurrence of schwannomatosis and rhabdoid tumor predisposition syndrome 1
Source: Mol Genet Genomic Med. 2018 May 20;6(4):627–37. doi: 10.1002/mgg3.412 (PMC6081224; doi:10.1002/mgg3.412)
Supplement: Supplementary file 1 [file MGG3-6-627-s001.pdf]

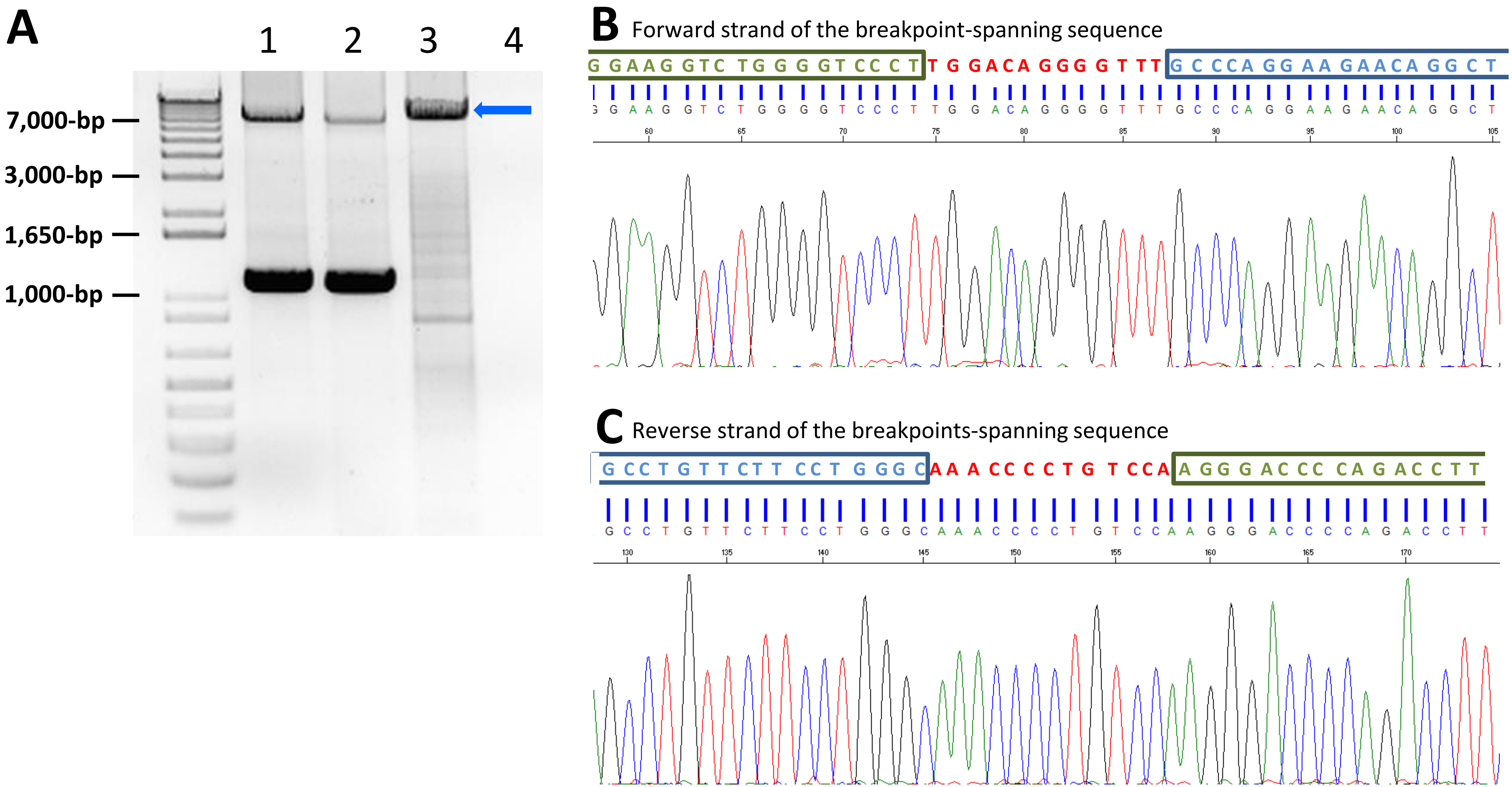

Supplementary Figure S1: Breakpoint-spanning PCR performed to identify the deletion junction of the germline *SMARCB1* deletion. (A) Gel-electrophoresis of PCR products amplified with primers US1_for10 and rev8 using blood-derived DNA of patient II.4 (lane 1), blood-derived DNA of the patient‘s mother, proband I.5 (lane 2), DNA isolated from blood of an unaffected donor without the *SMARCB1* deletion (lane 3) and with water used as PCR template (lane 4). The blue arrow indicates the 7,526-bp PCR fragment amplified from the chromosome 22 without the *SMARCB1* deletion. The breakpoint-spanning PCR fragment encompassing 1,138-bp was amplified exclusively from DNA of the patient and her mother but not from DNA of the unaffected donor. (**B**) Breakpoint-spanning sequence obtained with primer US1 used for the sequence analysis of PCR product US1_for10/rev8 amplified from blood-derived DNA of patient II.4. The insertion of 13-bp identified at the deletion junction is indicated in red, proximal sequences are indicated in green, whereas distal sequences are given in blue. (**C**) Sequence of the reverse strand of the breakpoint-spanning fragment obtained with primer rev13 used for the sequence analysis of PCR product US1_for10/rev8 amplified from blood-derived DNA of patient II.4.

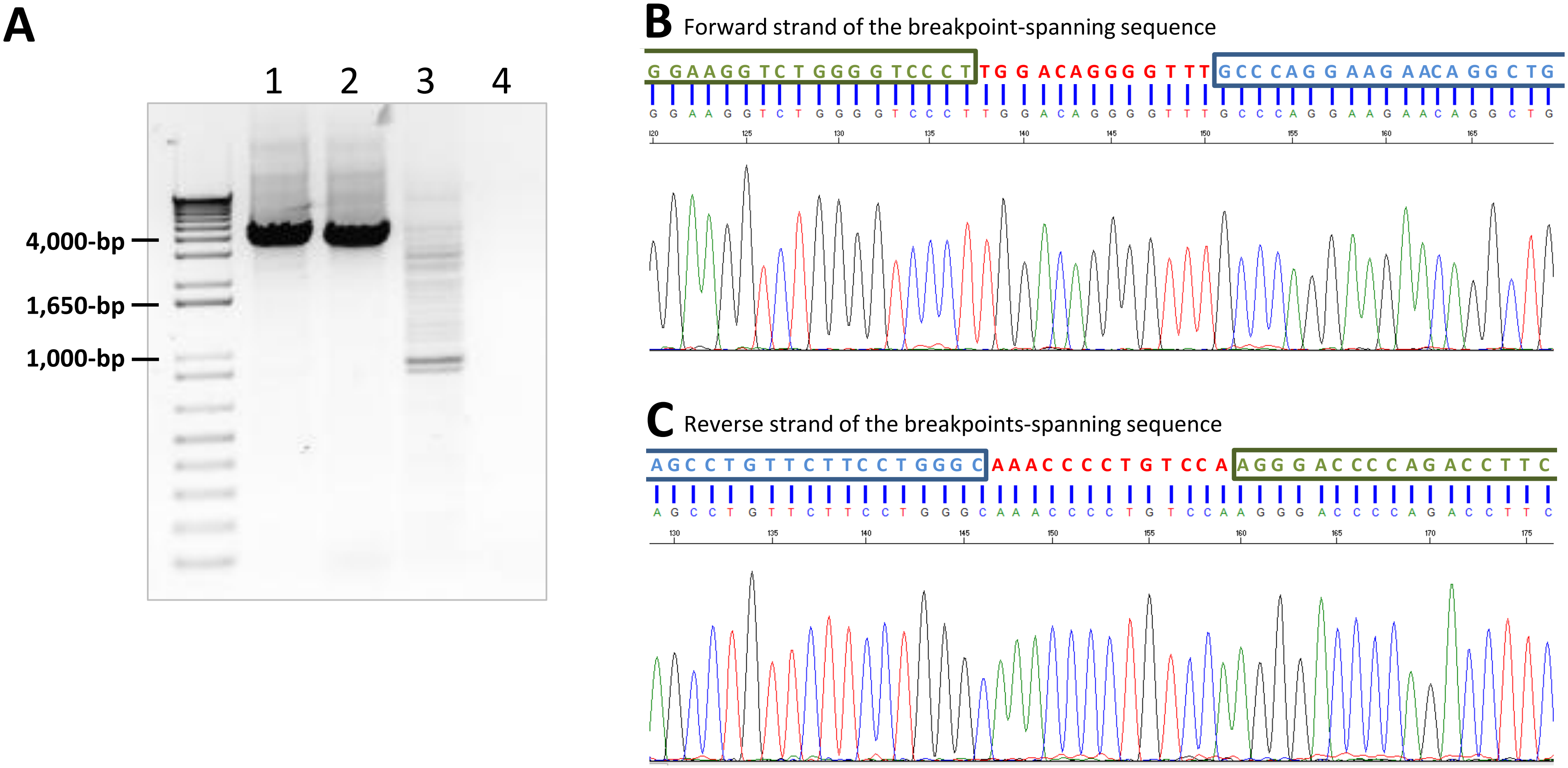

Supplementary Figure S2: Breakpoint-spanning PCR performed to identify the deletion junction of the germline *SMARCB1* deletion. (**A)** Gel-electrophoresis of PCR products amplified with primers using blood-derived DNA of patient II.4 (lane 1), blood-derived DNA of the patient‘s mother, proband I.5 (lane 2), DNA isolated from blood of an unaffected donor without the *SMARCB1* deletion (lane 3) and with water used as PCR template (lane 4). The 3,756-bp breakpoint-spanning PCR fragment was exclusively amplified from DNA of patient II.4 and her mother but not from the unaffected control individual. Complete sequence analysis of the 3,756-bp breakpoint-spanning PCR fragment derived from patient II.4 and her mother did not indicate any sequence differences between both individuals. (**B**) Breakpoint-spanning sequence obtained with primer for14 used for the sequence analysis of PCR product for9/rev8 amplified from blood-derived DNA of patient II.4. The insertion of 13-bp identified at the deletion junction is indicated in red. Proximal sequences are indicated in green whereas sequences located in telomeric direction to the insertion are indicated in blue. (**C**) Reverse sequence of the breakpoint-spanning fragment obtained with primer rev10 used for the sequence analysis of PCR product for9/rev8 amplified from blood-derived DNA of patient II.4.

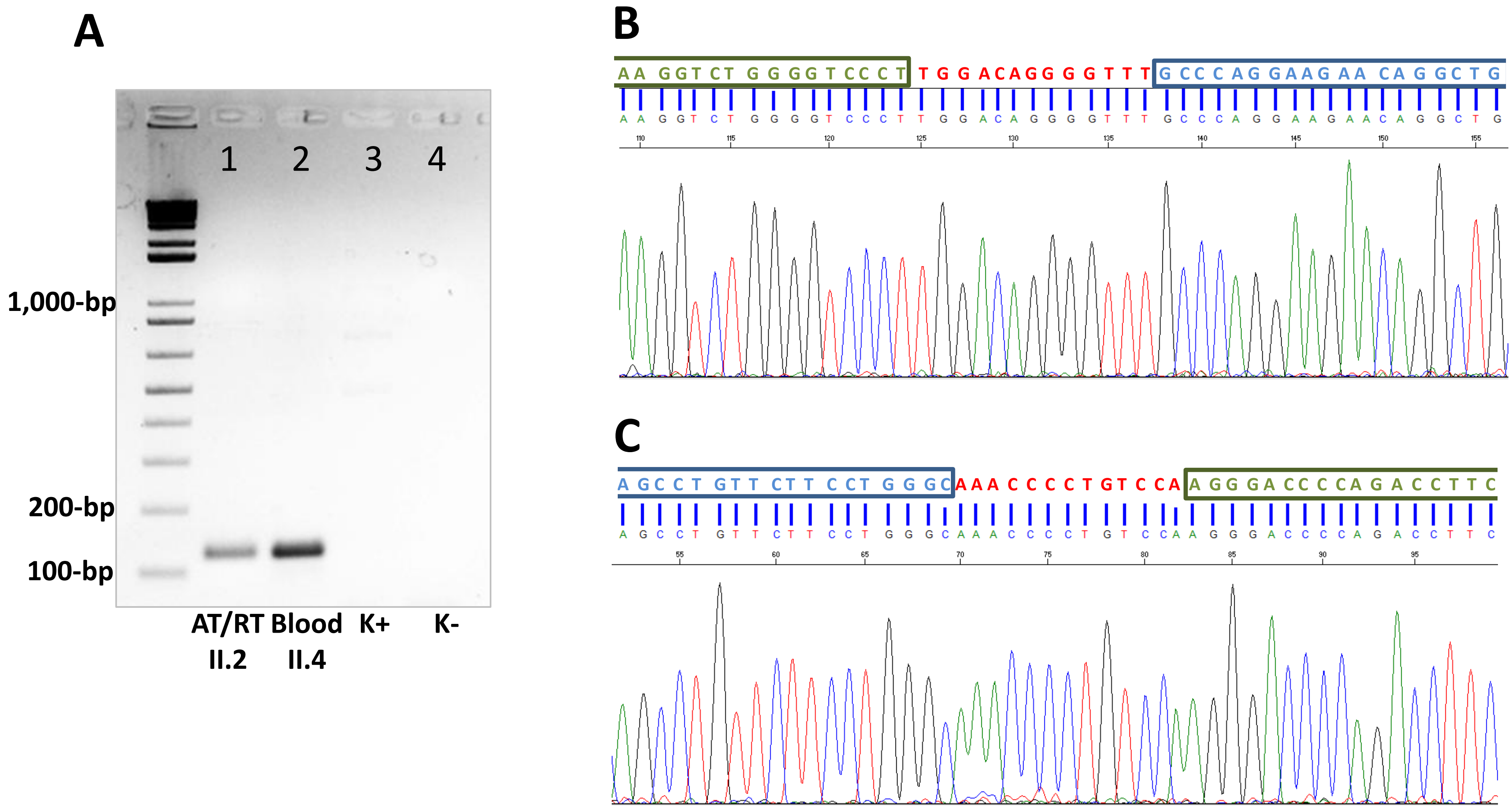

Supplementary Figure S3: Breakpoint-spanning PCR performed to identify the deletion junction of the germline *SMARCB1* deletion. (**A**) Gel-electrophoresis of PCR products amplified with primers US1_for10 and US2_rev13 using DNA isolated from paraffin-embedded AT/RT tissue of patient II.2 (lane 1), blood-derived DNA of patient II.4 (lane 2), DNA isolated from blood of a unaffected donor without the *SMARCB1* deletion (lane 3) and with water used as PCR template (lane 4). The breakpoint-spanning PCR fragment amplified from AT/RT-derived DNA of patient II.2 with primers US1_for10 and US2_rev13 was cloned and sequenced on the forward strand with vector-based primer T3 (**B**) and on the reverse strand with primer T7 (**C**). The insertion of 13-bp identified at the deletion junction is indicated in red.

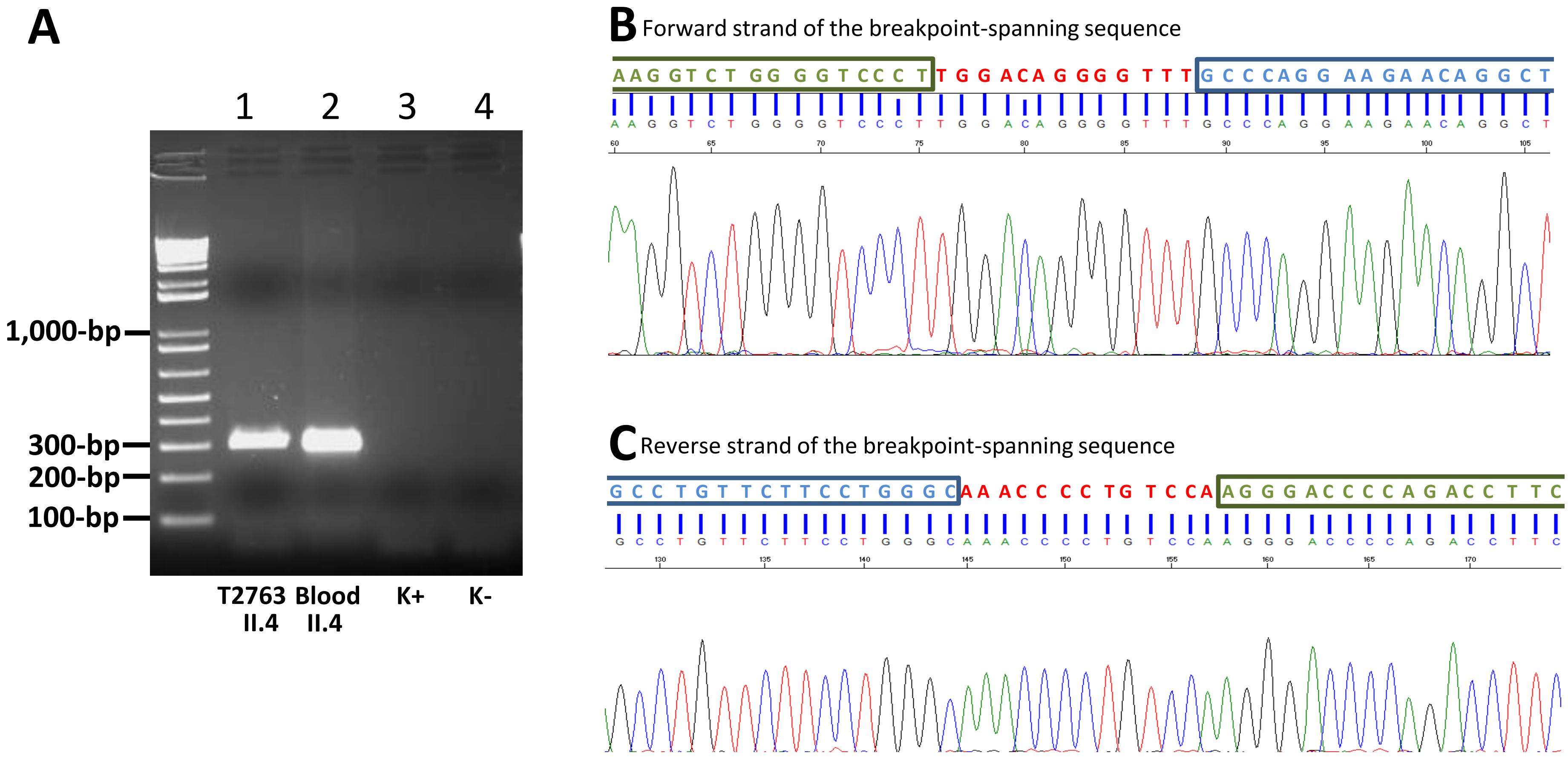

Supplementary Figure S4: Breakpoint-spanning PCR performed to amplify the deletion junction of the germline *SMARCB1* deletion. (**A**) Gel-electrophoresis of PCR products amplified with primers US1_for10 and rev10 using DNA isolated from schwannoma tissue (T2763) of patient II.4 (lane 1), blood-derived DNA of the patient II.4 (lane 2), DNA isolated from blood of an unaffected control individual without the *SMARCB1* deletion (lane 3) and with water used as PCR template (lane 4). (**B**) Breakpoint-spanning sequence obtained with primer US1 and primer rev10 (**C**) used for the sequence analysis of PCR product US1_for10/rev10 amplified from schwannoma-derived DNA of patient II.4. The insertion of 13-bp identified at the deletion junction is indicated in red.

Schwannoma 2763

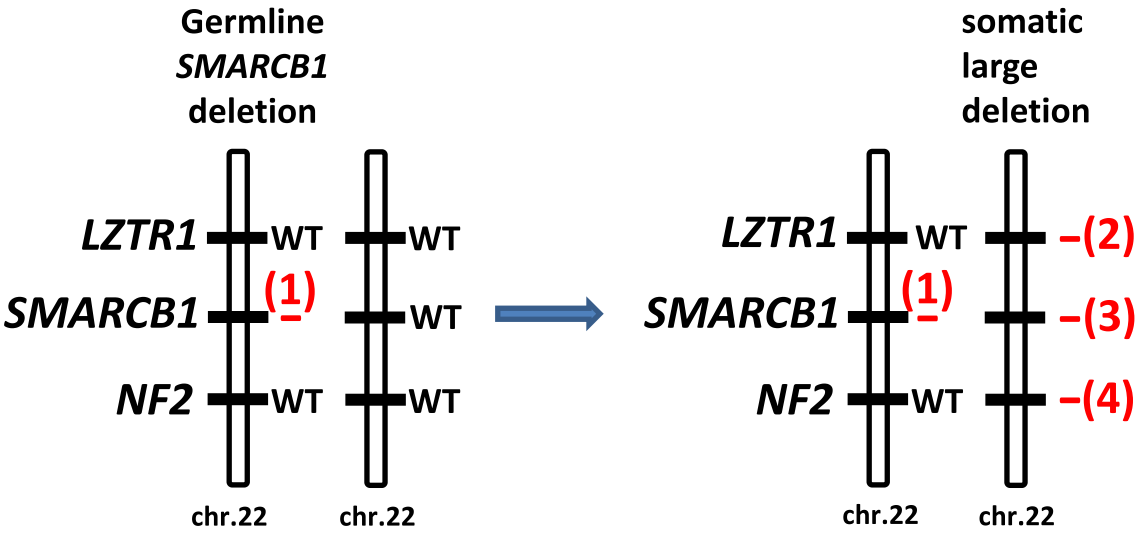

Supplementary Figure S5: Genetic changes detected in schwannoma T2763 of patient II.4. The germline *SMARCB1* deletion represents the first hit (1). This deletion encompasses 6,388-bp including parts of *SMARCB1* intron 7, exons 8-9 and 3,302-bp located telomeric to exon 9 including the 3' UTR of *SMARCB1*. The deletion junction of this germline *SMARCB1* deletion was amplified from schwannoma DNA as determined by deletion breakpoint-spanning PCR (Supplementary Figure S4). MLPA analysis of genomic DNA isolated from schwannoma T2763 tissue indicated the somatic loss of one copy of *LZTR1*and *NF2* which was not detected in the patient's blood. Hence, a large deletion must have occurred on the chromosome 22 not harbouring the germline *SMARCB1* deletion of exons 8 and 9. This somatic deletion caused the loss of one allele of *LZTR1*, *NF2* and the wildtype allele of *SMARCB1* (hits 2-4). Chr.22: Chromosome 22.
