# Supplementary material for: Co‐occurrence of schwannomatosis and rhabdoid tumor predisposition syndrome 1
Source: Mol Genet Genomic Med. 2018 May 20;6(4):627–37. doi: 10.1002/mgg3.412 (PMC6081224; doi:10.1002/mgg3.412)
Supplement: Supplementary file 2 [file MGG3-6-627-s002.docx]

**Supplementary Table S1:** Sequence of the primers used for breakpoint-spanning PCRs (marked in blue). Primers for9 and rev8 were also used for the sequence analysis of the breakpoint-spanning PCR products. Additional sequencing primers are marked in grey. US1 is a universal primer used as sequencing primer.

| **Position of the primer** | **Primer ID** | **Sequence 5´→3´** | **Position** | **T_A_ [°C]^a^** |
| --- | --- | --- | --- | --- |
| *SMARCB1* intron 7 | for9 | GCTGATTCCCCAAGAGCCAGT C | 24,170,890 – 24,170,911 | 62 |
| *SMARCB1* intron 7 | for11 | CACACCAGCAGTCCTACACGTAC | 24,171,617 – 24,171,639 | 62 |
| *SMARCB1* intron 7 | for12 | CCAGGAGTAGAATTGCTGGGTCAC | 24,172,358 – 24,172,381 | 62 |
| *SMARCB1* intron 7 | rev12 | GTGAAAAGGCAATCCTCCAAATGAGAG | 24,172,751 – 24,172,777 | 61 |
| *SMARCB1* intron 7 | US1_for10 | GTAGCGCGACGGCCAGTCTCTGTCCCTCAGACGTGAGCATC | 24,173,530–24,173,553 | 62 |
| *SMARCB1* intron 7 | rev14 | GATGCTCACGTCTGAGGGACAGAG | 24,173,530 – 24,173,553 | 62 |
| distal to *SMARCB1* exon 9 | rev10 | GTCGCCTTTATTGGACTGGCTCATC | 24,180,159 – 24,180,183 | 62 |
| distal to *SMARCB1* exon 9 | for3 | GATGAGCCAGTCCAATAAAGGCGAC | 24,180,159 – 24,180,183 | 62 |
| distal to *SMARCB1* exon 9 | rev11 | CAAAGCCATGCACAAAAGAGCCAG | 24,180,325 – 24,180,348 | 62 |
| distal to *SMARCB1* exon 9 | rev8 | CGCCTTGGTTACAGCCTCTGG | 24,181,036 –24,181,056 | 62 |
| distal to *SMARCB1* exon 9 | rev13 | CTGGGCAGCCTGTTCTTCCTG | 24,180,009-24,180,029 | 61 |
| *SMARCB1* intron7 | for14 | GTGTGTTGCATCACATGAGGTCATG | 24,173,445-24,173,469 | 60 |
|  | US1 | GTAGCGCGACGGCCAGT |  | 55 |

a: T_A_, annealing temperature

**Supplementary Table S2**: Sequence of the primers US1_for10 and US2_rev13 used for the breakpoint-spanning PCR with DNA isolated from paraffin-embedded tissue of the AT/RT of patient II.2. PCR product US1_for10/US2_rev13 was cloned by means of the StrataClone PCR cloning kit (Agilent) and sequenced with vector-based primers T3 and T7.
Primers US1_for10 and rev10 were used to amplify a breakpoint-spanning PCR product from genomic DNA isolated from schwannoma T2763 of patient II.4. PCR product US1_for10/rev10 was sequenced using primers US1 and rev10.

| **Position of the primer** | **Primer ID** | **Sequence 5´→3´** | **Position** | **T_A_ [°C]** |
| --- | --- | --- | --- | --- |
| *SMARCB1*  intron 7 | US1_for10 | GTAGCGCGACGGCCAGTCTCTGTCCCT CAGACGTGAGCATC | 24,173,530 – 24,173,553 | 62 |
| distal to *SMARCB1*  exon 9 | US2_rev13 | CAGGGCGCAGCGATGACCTGGGCAGC  CTGTTCTTCCTG | 24180009 – 24180029 | 61 |
| distal to *SMARCB1*  exon 9 | rev10 | GTCGCCTTTATTGGACTGGCTCATC | 24180159 – 24180183 | 60 |
|  | US1 | GTAGCGCGACGGCCAGT |  | 55 |
|  | T3 | GCAATTAACCCTCACTAAAGG |  | 57 |
|  | T7 | TAATACGACTGACTATAGGG |  | 57 |

a: T_A_, annealing temperature

**Supplementary Table S3:** Sequence of the primers used for the amplification of the LZTR1 exons from blood-derived DNA of the patient II.4. At the 5’-end of each forward primer (for) the sequence tag 5’-GTAGCGCGACGGCCAGT-3’ was added and at the 5’-end of each reverse primer (rev) the sequence tag 5’-CAGGGCGCAGCGATGAC-3’. These sequence tags served as binding sites for the universal sequencing primers US1 (5’-GTAGCGCGACGGCCAGT-3’) and US2 (5’-CAGGGCGCAGCGATGAC-3’).

| ***LZTR1* exon** | **Primer ID** | **Sequence 5´→3´** | **Position (hg19)** | **T_A_** |
| --- | --- | --- | --- | --- |
| 1 | Exon 1 for | GGACGACACACTGCATTCACTG | 21,336,367-21,336,388 | 59 |
|  | Exon 1 rev | GACTCCCTCGGAACCCTCTC | 21,337,012-21,337,031 | 59 |
| 2 | Exon 2 for | GCTCTCCTGCTTAGTCCCAT | 21,337,237-21,337,256 | 57 |
|  | Exon 2 rev | GGACAGTAATGGAGCTGGACA | 21,337,445-21,337,465 | 57 |
| 3 | Exon 3 for | GAGCCTCCTTTGTGGTCACC | 21,339,954-21,339,973 | 58 |
|  | Exon 3 rev | CTTGATCTCCTCAGCCTGACAG | 21,340,349-21,340,370 | 58 |
| 4 | Exon 4 for | GTGTGGACCTCATGGGTGACC | 21,341,737-21,341,757 | 59 |
|  | Exon 4 rev | GCAGAAGGGCAGGGTGTC | 21,341,921-21,341,939 | 59 |
| 5 | Exon 5 for | CCAGATTCTGCTCCACCTTCCAGG | 21,342,217-21,342,240 | 59 |
|  | Exon 5 rev | CACCAATCCCAAGCTCCCTG | 21,342,517-21,342,536 | 59 |
| 6 | Exon 6 for | GCCTTGTGGAGGTCCTGAAG | 21,342,934-21,342,953 | 58 |
|  | Exon 6 rev | CCAGCCTAAGCCCTCAAATGC | 21,343,228-21,343,248 | 58 |
| 7 | Exon 7 for | GCCATCCCTTCCAGCCAG | 21,343,837-21,343,851 | 63 |
|  | Exon 7 rev | GAAGAAAGCAGCCTCGACCC | 21,344,110-21,344,129 | 63 |
| 8 | Exon 8 for | CCGTGAAGTGGATGAGACAGG | 21,344,504-21,344,524 | 58 |
|  | Exon 8 rev | CTGTAACCTCCTGCTGTTTGCAGAG | 21,344,925-21,344,949 | 58 |
| 9 | Exon 9 for | CTGGTGCCCGTGCTGGATG | 21,345,733-21,345,751 | 60 |
|  | Exon 9 rev | GCCTTGCTCCTGGTAGCTGTC | 21,346,221-21,346,241 | 60 |
| 10 | Exon 10 for | CCATGCAGCTCTTCCTTCTTTCAG | 21,346,387-21,346,410 | 58 |
|  | Exon 10 rev | GTGGCACCCACCCCAGAAAG | 21,346,825-21,346,844 | 58 |
| 11 | Exon 11 for | CCCACCTGTGTCTGTACCCA | 21,346,923-21,346,942 | 59 |
|  | Exon 11 rev | CAAGGCTTCCATCCTGCCAG | 21,347,305-21,347,324 | 59 |
| 12 | Exon 12 for | GTGGACAGTTGCAGGTGCTG | 21,347,744-21,347,763 | 62 |
|  | Exon 12 rev | CAAAGAATGATGCACTGGCAGAGG | 21,348,184-21,348,207 | 62 |
| 13/14 | Exon 13/14 for | CTGACCTTGGCTGGCTGG | 21,348,135-21,348,152 | 61 |
|  | Exon 13/14 rev | GCCACTAGGTAGGAACAAGGC | 21,348,733-21,348,753 | 61 |
| 15 | Exon 15 for | CCA GCC CAC ACT CTT CCA TG | 21,348,755-21,348,774 | 58 |
|  | Exon 15 rev | GAAGTTCAGGCAGTGCTCCTG | 21,349,156-21,349,176 | 58 |
| 16 | Exon 16 for | GCTGAGCCAACTCAAGGTGTGG | 21,349,001-21,349,022 | 60 |
|  | Exon 16 rev | CCTGTACCCAGGAGAGCACTCTG | 21,349,439-21,349,461 | 60 |
| 17/18 | Exon 17/18 for | GGCAGCAACATGGGCAGATA | 21,349,939-21,349,958 | 59 |
|  | Exon 17/18 rev | GGCAGTTGTGAGGGTCAGGA | 21,350,618-21,350,637 | 59 |
| 19/20 | Exon 19/20 for | CCCTTCCCTGTCCTTCCCT | 21,350,912-21,350,931 | 58 |
|  | Exon 19/20 rev | GGCTGCTCTGCTTCTCTCAC | 21,351,365-21,351,384 | 58 |
| 21 | Exon 21 for | GTGAGAGAAGCAGAGCAGCC | 21,351,365-21,351,384 | 59 |
|  | Exon 21 rev | CCTCGCATCCACACTACACCAG | 21,351,881-21,351,902 | 59 |

T_A_: annealing temperature in °C

**Supplementary Table S4**: Sequence of the primers used for the amplification and the sequencing of the *NF2* gene exons. Primers E6_2 rev and E9_2 rev were only used for sequencing whereas primers E6 rev and E9 rev were only used for amplification of PCR products. The annealing temperature for all PCRs and sequencing reactions was 60°C.

| ***NF2* exon** | **Primer ID** | **Sequence 5´→3´** | **Genomic postion (hg19)** |
| --- | --- | --- | --- |
| 1 | E1 for | GGCTAAAGGGCTCAGAGTGC | 29,999,927-29,999,946 |
|  | E1 rev | CTTCCACCTCGACTGTCACC | 30,000,130-30,000,149 |
| 2 | E2 for | GGAACCTGAGAGTGGAGAGTGC | 30,032,654-30,032,675 |
|  | E2 rev | CTGGAAAGCTCACGTCAGCC | 30,032,895-30,032,915 |
| 3 | E3 for | GGTAGCACAGGAGGAAGTGC | 30,035,020-30,035,039 |
|  | E3 rev | CCATTCTTCACCATCACACCTTCAC | 30,035,405-30,035,429 |
| 4 | E4 for | CTCATTAGAACGCCGTGAGGC | 30,038,097-30,038,117 |
|  | E4 rev | CAAGTCCACAAGTCCCATAACATCTCAC | 30,038,389-30,038,416 |
| 5 | E5 for | GCAAGGTTGAAATCTTGACTTGTCATCGG | 30,050,507-30,050,535 |
|  | E5 rev | CAGAGATCCCACACTGTTTACTGGAGATTAC | 30,050,835-30,050,865 |
| 6 | E6 for | GTGACTATCTCCCTGGGTGTAGC | 30,051,447-30,051,469 |
|  | E6 rev | GCATGTCCTAGTTTTGCAGTTCTTTAAGG | 30,051,723-30,051,751 |
|  | E6_2 rev | GCCCATAAAGGAATGTAAACCAAC | 30,051,682-30,051,705 |
| 7 | E7 for | GAGCTTTGCTGGTGTCTGCTG | 30,054,028-30,054,048 |
|  | E7 rev | GAGTCTATCGCCTTGGAATGAAGAAGTAAC | 30,054,324-30,054,353 |
| 8 | E8 for | CCCAATTCAGAAAGCTGAAATCTTCTTGG | 30,057,025-30,057,053 |
|  | E8 rev | CAAGGAGATGCCCTGGCTG | 30,0574,00-30,057,418 |
| 9 | E9 for | GAAGCCAGGACAAGGCATAACTTC | 30,060,852-30,060,875 |
|  | E9 rev | GCTGAGCCAGGAGAATCTCTTGAATC | 30,061,327-30,061,352 |
|  | E9_2 rev | GCTAAACAGAAAGTATGCGCCAAG | 30,061,167-30,061,190 |
| 10 | E10 for | GCATTCATCTTCACGTTTACTGCTACC | 30,064,206-30,064,230 |
|  | E10 rev | CAAGAGCAGGTATGTCCACGG | 30,064,531-30,064,551 |
| 11 | E11 for | GGCAATAAGAATGACCCTGGCTAC | 30,067,666-30,067,689 |
|  | E11 rev | GCAAACGCTGCTAACTCATGG | 30,067,666-30,068,036 |
| 12 | E12 for | CAGGAGTCCGAGACTCTGGTTTG | 30,069,154-30,069,176 |
|  | E12 rev | GTCACCATCAGCCAAGGAGGAG | 30,069,603-30,069,624 |
| 13 | E13 for | CCTCTTTGGGTGCCATCCTC | 30,070,707-30,070,726 |
|  | E13 rev | GCCATGTGTTTGCCTGAATGGTC | 30,071,025-30,071,047 |
| 14 | E14 for | CTGTGGCTGCTGGAGGATC | 30,074,094-30,074,112 |
|  | E14 rev | GGAGTAATGACAGGCTGCTGC | 30,074,405-30,074,425 |
| 15 | E15 for | CTCAAACCCTAGATCGCACACC | 30,077,323-30,077,344 |
|  | E15 rev | GTCTAGTGACTTGGCTACTGAGGAAAC | 30,077,661-30,077,687 |
| 16 | E16 for | GCAGATGGCACTTATGGCATTGTTG | 30,078,934-30,078,958 |
|  | E16 rev | CCTCGTAAGGGCTCAGTGTGG | 30,079,145-30,079,165 |
| 17 | E17 for | GACTGACAGCCAACTTCTTGAGC | 30,090,591-30,090,613 |
|  | E17 rev | CTCTACAGGGTCGTAGTTCAAGGC | 30,090,958-30,090,981 |
